# Leveraging network structure in nonlinear control

**DOI:** 10.1038/s41540-022-00249-2

**Published:** 2022-10-01

**Authors:** Jordan Rozum, Réka Albert

**Affiliations:** 1 Department of Physics, Pennsylvania State University, University Park, PA 16802 USA; 2 Department of Biology, Pennsylvania State University, University Park, PA 16802 USA

**Keywords:** Control theory, Regulatory networks

## Abstract

Over the last twenty years, dynamic modeling of biomolecular networks has exploded in popularity. Many of the classical tools for understanding dynamical systems are unwieldy in the highly nonlinear, poorly constrained, high-dimensional systems that often arise from these modeling efforts. Understanding complex biological systems is greatly facilitated by purpose-built methods that leverage common features of such models, such as local monotonicity, interaction graph sparsity, and sigmoidal kinetics. Here, we review methods for controlling the systems of ordinary differential equations used to model biomolecular networks. We focus on methods that make use of the structure of the network of interactions to help inform, which variables to target for control, and highlight the computational and experimental advantages of such approaches. We also discuss the importance of nonperturbative methods in biomedical and experimental molecular biology applications, where finely tuned interventions can be difficult to implement. It is well known that feedback loops, and positive feedback loops in particular, play a major determining role in the dynamics of biomolecular networks. In many of the methods we cover here, control over system trajectories is realized by overriding the behavior of key feedback loops.

## Introduction

Modeling the dynamics of complex biological systems has important implications for the understanding of fundamental biological processes, for exploring the effects of mutations, and for the development of new drugs. Aside from applications to practical problems in biology and medicine, studying the dynamics of large, complex networks (systems with many and varied types of interactions) raises unique and interesting mathematical challenges that separate it from the study of traditional dynamical problems in typically lower dimensions.

Perhaps the most impactful of these challenges is one of scale. These dynamical systems typically exist in state spaces with dozens or even hundreds of variables. Not only does this create computational hurdles, it also creates conceptual ones. Many qualitative techniques, such as phase portrait analysis, become impractical or even impossible at this scale. At a more mathematically fundamental level, the complex networks in biology typically come equipped with a privileged set of coordinates, i.e., the coordinates that are specified by the underlying network structure. This is in contrast to many physical systems, where coordinates are simply designators for points in phase space, and one is often free to choose coordinates for analytical or computational convenience. In biomolecular networks, however, the coordinates correspond to specific genes and proteins, and they therefore have inherent meaning. This distinction is important in designing control interventions because interventions that affect a smaller number of variables are more practical, and thus more desirable than those that affect many variables.

The dynamics of biological networks are almost always highly nonlinear. Classical results that apply exactly to linear systems can only be applied locally and require perturbative extensions. In the case of designing a control intervention, a perturbative extension entails applying a control to a linearization of the system at its current state to make a small perturbation in the desired direction in the state space. A new state is achieved, and the system is linearized at this new location; the process repeats, often with no a priori guarantee that the desired target state is actually reachable. This perturbative approach is mathematically elegant, but its application is limited to cases in which a control variable can be finely controlled. In contrast, nonperturbative methods can account for the nonlinearity of the system and can be applied when signals cannot be fine-tuned. These approaches are not without their own limitations; typically, they focus only on attractor control, wherein the goal is to drive a system into one of its attractors, rather than to an arbitrary state. There are relatively few nonperturbative methods available. There are even fewer nonperturbative approaches to dynamical control that can account for the nonlinearity of the system and also remain practical in high-dimensional state spaces. Those that do exist rely heavily on the relationship between the dynamics and the underlying interaction network. This interaction network may be viewed as the edge-signed directed graph whose adjacency matrix is constructed from the signs of entries in the Jacobian matrix. A value of *+1* in the *i, j* entry indicates that the *i*^*th*^ variable (e.g., gene or protein) has an activating effect on the *j*^*th*^ variable (e.g., gene). Similarly, a value of *−1* indicates an inhibitory effect. For general systems, the signs of the Jacobian matrix’ entries do not have fixed signs, but in biological systems, individual regulatory effects are almost universally monotonic, or else the state space can be appropriately partitioned into regions in which each entry of the Jacobian matrix is of constant sign^1^. In other words, an edge in a network almost always corresponds to a strictly inhibitory effect or a strictly activating effect for all points in configuration space. This constraint underlies several powerful and elegant results about the dynamics on such networks.

In addition to the above mathematical considerations, there are important practical considerations to keep in mind. First, biological data are often characterized by a large degree of noise and uncertainty in parameter values or even in functional forms^2^. This increases the desirability of control methods that are agnostic to the fine details of the regulatory effects. Such methods are often called “structural” because they rely primarily (or even entirely) on the structure of the underlying network; we discuss several such methods in this review. In addition, many biological systems are such that fine control over interventions is not practical or, sometimes, not possible at all. Experimentally, it is often only possible to knock out or knock in (overexpress) a gene. In contrast, classical control theory has been historically applied to systems in which the investigator can exert precise control over the components of a low-dimensional system. As such, much effort has been dedicated to the question of how to finely engineer these inputs to efficiently achieve a desired goal. In the context of biological network control the question rather is how to select controllable elements that can steer the system to a desired state using the “blunt instruments” of complete knockout and extreme overexpression. Furthermore, interventions in biological systems often occur on time scales that are much longer than the dynamics they are intended to affect. This especially complicates the application of perturbative control methods.

This review focuses on methods of dynamically controlling complex biological networks with an emphasis on nonperturbative, nonlinear methods that explicitly leverage network structure. We highlight the successes of bespoke methods that specifically ameliorate the unique challenges these systems pose while leveraging their unique properties. We discuss how regulatory feedback plays a crucial role in determining the attractor repertoire of these systems, and how this can be exploited by control methods. We emphasize that these control methods must be considered in the context of the network-specified coordinates when evaluating the viability of their outputs as real-world interventions.

## Challenges of network control

### High dimension, observability, and visualization

Dynamical models of biological systems typically exist in state spaces with dozens or even hundreds of variables. For example, Wittmann et al.^3^ present a 29-dimensional Hill kinetics ODE model of T-cell signaling; von Dassow and collaborators^4,5^ present a model of *Drosophila* segment polarity with 50 free parameters and 8 genes and proteins per cell in an arbitrarily sized 2-dimensional hexagonal grid; and Chen et al.^6^ present a model of the cell cycle in budding yeast that involves 39 independent state variables and 97 free parameters. Many qualitative techniques, such as phase portrait analysis, become impractical or even impossible for such a high number of variables. Furthermore, it is usually the case that the dimension of the parameter space scales alongside the dimension of the state space. This has encouraged a great deal of effort in developing parsimonious models that use as few variables as possible to describe a certain set of behaviors. It is partly for this reason that ODE and PDE models of biological systems often have many fewer variables than discrete models of the same systems. For example, a pioneering ODE model of TGF-β-induced epithelial to mesenchymal transition^7^, published in 2014, has eight variables and 15 interactions. A Boolean model of the same process, published in the same year, has 70 variables and 135 interactions^8^ and predicts that autocrine and paracrine signaling leads to crosstalk between pathways that were previously thought to be independent. Several years later, the importance of much of the crosstalk between pathways in TGFβ-induced mesenchymal transitions was verified experimentally^9^.

Interpreting the results of numerical simulations also becomes fraught, as one must judiciously select which variables are most important for validating the model and predicting the system state from observations. Often, success requires the artful application of domain expertise. Typically, variables that correspond to known biological markers are selected for consideration in these models. This is done partly because these are already known to be important in the system of interest, but it is also done because data about key markers are more readily available. As a practical matter, most biological data is taken in or near a system’s attractors; it is often the case that the majority of the variables are fixed in the attractors of biological models, which allows one to characterize most or all of the variables precisely. Sometimes, however, transient behaviors are of interest. In these cases, it is desirable to identify a set of observables that fully characterizes any unique system trajectory. See, e.g., refs. ^10–12^ for further discussion of the so-called “observability” problem and ref. ^13^ for discussion of the related problem of “structural identifiability” which considers identifying the values of static parameters. One must be careful to identify which variables must be observed in order to determine the state of the system; this is necessary both for validating a model and also for applying a model to uncover new biological insights. There have also been cases when focusing only on previously known key markers might have missed important dynamical properties of the system. For example, the crosstalk between various pathways during the epithelial to mesenchymal transition predicted by Steinway et al.^8^ and confirmed by Deshmukh et al. ^9^ is not observable from the core regulatory circuitry alone.

### Uncertainty in dynamic specification

The challenges of high dimension are compounded by the related challenge of uncertainty in the specification of the dynamics. In many protein-protein and protein-DNA interactions, kinetic parameters are poorly constrained by the available data, with parameter regions often spanning multiple orders of magnitude^4^. Even more extremely, often the functional form of the regulatory dynamics is unknown^2^. Despite the fact that biomolecular network models are typically robust to some degree of uncertainty, these uncertainties can nevertheless be large enough to have profound effects on the dynamics^2,14^. High dimension makes many traditional approaches to studying bifurcation impractical. For example, the straightforward approach is to analyze the number of roots in the Jacobian characteristic polynomial and the number of steady states as functions of parameters. Unfortunately, there is no sufficiently fast method to solve the large systems of nonlinear equations that result when these methods are applied to high-dimensional ODEs with many parameters.

A common approach in such systems is to fix a functional form and to specify a reasonable set of parameters to vary. The parameter values are often fit heuristically (e.g., through the use of a genetic algorithm^15^). This is often followed by parameter robustness tests. Fortunately, robustness to changes in the values of parameters is a common feature of biomolecular systems, making this approach much more feasible than one might expect^4,16,17^.

### The nonlinear and nonperturbative nature of experimental probes

Classical control theory was initially developed for applications outside molecular biology and genetics (see Macki and Strauss^18^ for an introduction to classical methods). In many of these applications, the researcher has a great deal of freedom to modify and tune feedback loops and external signals. The traditional mathematical theory and techniques reflect this–many control methods for network dynamics consider the effects of arbitrary time-varying control inputs that linearly modulate the dynamics, i.e., equations of the form

1 $$\frac{{dx}}{{dt}} = f\left( x \right) + G\left( x \right)u\left( t \right)$$

where *x* is the *N*-dimensional state vector, *f* is the regulatory function, and *u*(*t*) is a vector of *M*control inputs modulated by the *N* *×* *M* matrix function *G(x)*. In general, the regulatory and control-mediation functions *f* and *G* may be nonlinear in *x*, but often some sort of linearization is considered, and the effects of *u(t)* are treated perturbatively. The linearization may be explicit, such as when dynamics near a specified trajectory or equilibrium are considered, or they may be implicit, such as when various Lie algebraic approaches are applied (in which case it is the action of the control that is implicitly linearized). We will briefly highlight several of these approaches; for a more detailed discussion, see the review by Liu and Barabasi^11^ or the textbook by Sontag^19^.

Many of the foundations and key early results in the study of perturbative control in nonlinear systems were put forth by Kalman in the 1960s. In particular, Kalman^20^ defines various necessary conditions for the controllability of nonlinear systems and presents the Kalman rank criterion, which describes conditions for the controllability of the system of Eq. (1) for $f\left( x \right) = Ax$ linear and *G* constant. Specifically, such a system is controllable if and only if the *N* *×* *NM* matrix *C* = [*G,* *AG,* *A*^2^ *G*,...,*A*^*N*−1^ *G*] has full rank. This result serves as the basis for so-called “structural controllability”, which extends the results of Kalman by considering only the structure of *A* and *G* as graph adjacency matrices and ignoring the precise values of their entries^21,22^.

Several Lie algebraic methods^11,19,23,24^ apply to Eq. (1) generally, and typically involve considering $f\left( x \right)$ and the columns of $G\left( x \right)$ as generators of a Lie algebra $L\left( {x_0} \right)$ at a point﻿ ${x_0}$. This correspondence between the columns of $G\left( x \right)$ and the generators of a Lie algebra can be obtained by assigning to each column vector the differential operator that computes the directional derivative in the direction of the column vector at each point *x*. The infinitesimal group action of $L\left( {x_0} \right)$ (or, equivalently, of the set of the directional derivatives in the direction of the columns of $G\left( x \right)$) on the state space at a point ${x_0}$ describes the set of locally accessible states in some neighborhood of ${x_0}$. Therefore, if the action of $L\left( {x_0} \right)$ does not span the full tangent space at ${x_0}$, there are states in a neighborhood of ${x_0}$ that are not locally accessible by the control inputs $u\left( t \right)$. Note that this is not equivalent to the statement that these states are not reachable eventually under any sequence of controls; perhaps it is possible to reach such states by moving far from ${x_0}$ to a point $x_1$, where the system is more easily manipulated, making a control adjustment, and returning to a point near *x*_0_ that was not locally accessible. Furthermore, even if $L\left( {x_0} \right)$ is full rank, it does not always guarantee controllability; for example, the action of a control may be unidirectional. A classic example, which we adapt from Liu and Barabasi^11^, is the system $\left( {\dot x,\dot y} \right) = \left( {y^2,u} \right)$. At $\left( {x_0,y_0} \right) = \left( {0,0} \right)$, the Lie algebra generated by the control is spanned by (0,u) and (1,0) and is thus full rank; however, states with negative values in the first component cannot be reached by this control. In general, determining whether a system is controllable is considerably more difficult than conducting local accessibility tests; the disparity in computational complexity is discussed in detail by Sontag^24^.

These perturbative methods, when applied to nonlinear systems, are generally concerned with identifying necessary conditions for full system control, i.e., for the ability to drive the system into an arbitrary state in finite time. This stands in contrast to many of the methods we will discuss here, in which the primary focus is attractor control: the ability to drive the system into a target attractor. This more limited focus allows one to circumvent some of the computational and experimental difficulties associated with more general questions of controllability^24^ while also making coarser (i.e., nonperturbative) control functions more viable.

Furthermore, in many biological applications, the precise control assumed by many traditional mathematical methods is not reasonably attainable. In many clinical settings, for example, drug dosage can only be manipulated on timescales on the order of hours – it is not feasible to instruct a patient to take drugs according to a complicated and frequent schedule. In experiments probing gene function, genes are often completely knocked out or forcibly overexpressed. The timing of these interventions can be controlled to some degree, e.g., using various genetic switches, but the magnitude of the effect is generally difficult to control. This is in part due to fundamental biological limitations stemming from the inherent nonlinearity of the systems. For example, activation of an auto-activating gene can be irreversible and its transcription rate is not easily modulated by external controls. In practice, this can limit the utility of powerful classical nonlinear control methods that identify inputs that can globally or locally control a system, such as control Lyapunov methods^19,25^, but which assume the ability to carefully engineer input signals. Despite the biotechnological challenge of implementing finely tuned control strategies, there have been notable successes in applying optogenetic methods to study and control cardiac processes^26^, neurological signaling^27^, molecular signaling pathways^28^, and gene expression^29^.

## Leveraging network structure

In this section, we will lay out a few common goals for analyses of complex network dynamics and discuss several ways in which researchers have leveraged network structure to accomplish them. Foremost among these aims is to fully characterize the attractor repertoire of the model so that individual attractors can be mapped to observed phenotypes. Attractors that do not correspond to any known phenotype may be spurious and suggest that further refinement of the model is needed. This refinement may take the form of structural changes to the underlying network, changes to the form of regulatory functions, or adjustments to parameter values. This last refinement is itself a key goal of many ODE models: to identify a reasonable set of parameters such that model outputs can be predictive. Translating model predictions into biologically meaningful experimental predictions is also nontrivial and requires careful identification of relevant biological markers – a task that often requires both domain expertise and can be aided by mathematical familiarity with the model itself. The final goal we will discuss is the identification of interventions that can drive the system toward a desired attractor or attractors.

A common theme in the analysis of complex network dynamics is identifying how a network’s structure constrains its dynamics^1,30–33^. In particular, it has been noted that feedback loops (paths of positive length that have the same starting and ending node) are responsible for much of the dynamic richness of biomolecular models. Sometimes feedback loops are analyzed within the context of auxiliary networks (e.g., in the graph structure of stoichiometric matrices; see Clarke^34^ for an overview), but often the analyses can be carried out directly using the interaction topology. The latter case is the focus of this section.

In a signed interaction network, any path or feedback loop (cycle) can be assigned a sign equal to the product of its edges’ signs. Positive and negative feedback loops have implications for the possible dynamical behaviors a system can exhibit. Many structural methods rely on a simple premise: in the absence of feedback loops, each variable’s behavior is determined entirely by the behaviors of its regulators. In this sense, the only nontrivial behaviors in the system must arise from feedback. This result can be made more specific or strengthened in various ways: in systems governed by sigmoidal kinetics, positive feedback is necessary for multistability and negative feedback is necessary for stable periodicity^35,36^ (see also work by Thomas and Kaufman^1,32^ for a discussion of implications and extensions); certain types of self-regulation do not induce nontrivial behaviors, an observation that gives rise to powerful techniques for attractor control^10,37^; and special feedback loops, called stable motifs, give rise to forward-invariant trap sets in the dynamics^38,39^.

This feedback-centric paradigm stands in contrast to some well-known results in the analysis of linear dynamics of complex networks. In particular, the structural controllability framework^21^, which is a graph-theoretic extension of the Kalman rank condition^20^, involves studying the branch points of the interaction topology while neglecting many of the feedback loops. While structural controllability and other linear methods have important consequences for perturbative, local control of nonlinear dynamical systems (or full control of linear systems) they can be ill-suited for studying how systems can be made to transition from one attractor to another. This primarily stems from the fact that feedback loops can give rise to dynamical trap sets that cannot be escaped using the methods of linear control alone^11,22^.

### Feedback vertex sets

A key result demonstrating the general importance of feedback in nonlinear systems is feedback vertex set (FVS) control^10,37^ and its generalization to account for external inputs^40^. A feedback vertex set is any set of nodes in a network whose removal would render the network acyclic. In general, there is not a unique smallest FVS. Typically, the number of minimal FVSs increases with the size and density of the network in question, and identifying a minimal FVS is an NP-complete problem. In practice, the computational burden of identifying a minimal FVS is negligible compared to the problem of numerically integrating the system, and for the methods discussed here, it is not strictly necessary that the FVS identified be minimal (though it is usually desirable). Observation of an FVS provides complete information about a trajectory. An FVS also has important applications in the control of these networks. In particular, by overriding the values of variables in an FVS to match with the values obtained in a specific attractor, one can guarantee convergence into that attractor. Notably, subject to mild boundedness criteria, this holds for any ODE that is consistent with the underlying interaction network, removing the need to fully specify the parameter values and functional forms in the regulatory functions. This flexibility has enabled successful applications to real biological systems^41,42^.

**Fig. 1:**
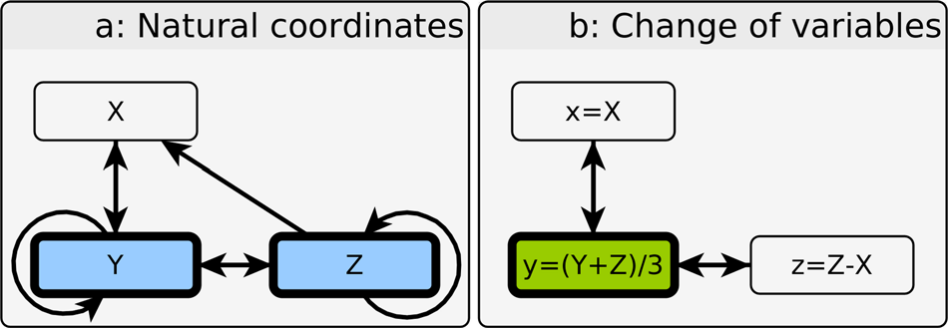
Network structure and minimal feedback vertex sets of Eqs. 2 and 3. In this example, Eq. 2 is written in a privileged, or natural set of coordinates that correspond to the activities of individual biomolecules. The minimal feedback vertex set of Eq. 2 is unique, and consists of variables *Y* and *Z*, highlighted in blue in the network on the left (panel a); in addition, all feedback vertex sets contain this as a subset due to the self-regulation of these two variables. This implies that overriding *Y* and *Z* is sufficient to attain either of the system’s two attractors. Furthermore, in this example, this control set is minimal. A change of variables (depicted in panel b) gives rise to a system with a smaller feedback vertex set, consisting only of a single variable, $y = \left( {Y + Z} \right)/3$, highlighted in green. However, in order to achieve the necessary override of *y* in the laboratory, *Y* and *Z* must both be manipulated. This example illustrates the importance of the natural coordinates of the system when designing control interventions.

It is important to recognize that changes of variables alter the topology of the interaction network, which has important implications for the interpretation of an FVS. Complex networks in biology typically come equipped with a privileged set of coordinates that correspond to specific genes and proteins; they have inherent meaning and experimental implications. This distinction is important to keep in mind when identifying observable sets: it is the number of original coordinate variables upon which the observables depend that is experimentally important, not the total number of observables. To illustrate this distinction, consider the following example ODE, which is analyzed in further detail in the Supplemental Notebook:

2 $$\begin{array}{l}\frac{dX}{dt} = H\left(\frac{Y+Z}{3}\right)-X\\ \frac{{dY}}{{dt}}=3H\left( X \right)H\left( {Z - X} \right)-Y\\ \frac{{dZ}}{{dt}}=2H\left( {\frac{{Y + Z}}{3}} \right) - Z,\end{array}$$

where $H\left( \cdot \right) = \left( \cdot \right)^4/\left( {\left( \cdot \right)^4 + 1/16} \right)$ is a Hill function. The wiring diagram of Eq. 2 is depicted in Fig. 1. Due to the small dimension of this example, it is straightforward to show that there are two stable steady states: $X = Y = Z = 0$, and $X \approx 1,Y \approx 4,Z \approx 2$ (there is also a single unstable steady state with $X \approx 0.06,Y \approx 0.07,Z \approx 0.12$). Here, the interaction graph is the complete digraph on three vertices minus one edge (*X* to *Z*) and with additional self-loops on *Y* and *Z* due to their autoactivation. Therefore, *Y* and *Z* constitute a minimal FVS (in this case the minimal FVS is unique). This implies that full attractor control of this system in a laboratory setting is guaranteed by controlling *Y* and *Z* together. In fact, this control set is minimal; setting any individual variable to zero fails to eliminate non-zero stable steady states in the evolution of the two free variables. This has experimental and clinical implications: if the desired behavior is to have all three variables inactive, any drug or knockout therapy must target both *Y* and *Z* directly – a task that in practice is more than twice as difficult than targeting only a single variable.

A change of variables, however, greatly simplifies the problem. Let $x = X$, $y = \left( {Y + Z} \right)/3$, and $z = Z - X$ to obtain

3 $$\frac{{dx}}{{dt}} = H\left( y \right) - x,\frac{{dy}}{{dt}} = H\left( x \right)H\left( z \right) - y,\frac{{dz}}{{dt}} = H\left( y \right) - z,$$

where *H* is defined as before. Here, the interaction graph has two cycles (see Fig. 1), $x \leftrightarrow y$, and $z \leftrightarrow y$, so *y* alone forms a minimal FVS (again, this is unique in this example). This implies that the control of *y* is sufficient to drive the system between its two steady states. Unfortunately, this elegant perspective on the controllability of the system ignores the fact that biological ODEs are more than the geometry of their vector fields – the real-world control problem remains difficult. That is not to say that such perspectives are never useful. In this particular example, the change of variables allows for an efficient way to find the system’s attractors because one need only vary *y* to search the state space for attractors. This observation underlies the methods of the next section.

### Monotone systems and subsystems

Interaction graphs are often equipped with signed edges, which indicate whether a particular entity encourages or inhibits the activity of another. Mathematically, these signed networks can be viewed as being constructed from the signs of entries in the Jacobian matrix (the matrix of first-order partial derivatives of the regulatory function), though as a practical matter, these structures are known with much more certainty than the mathematical forms that would give rise to any particular Jacobian. It is also customary to disregard constant negative terms along the diagonal of the Jacobian such that the interaction graph does not treat exponential degradation as autoinhibition. In some applications, strictly negative diagonal entries are disregarded entirely^43–45^.

In a sign-consistent network, all paths between each pair of nodes have the same sign. Such networks (disregarding any potential negative self-loops) yield monotone dynamics^43,44^. For a review of monotone dynamics, see Smith^46^. A key property of these systems is described in Kamke’s theorem, which states that in a sign-consistent network, perturbatively increasing a variable’s value always increases or decreases the values of other variables according to the sign of the paths between the perturbed and observed variable; furthermore, the propagation of this perturbation persists for all future times^45,46^. The analysis of so-called Monotone Input-Output Systems (MIOS) is facilitated by the construction of a characteristic input-output function. The construction of such a function relies on identifying an FVS of the sign-consistent network and creating a map between fixed constant values of the FVS variables and the values that their regulatory functions take in attractors of the controlled system. A precise definition is given by Angeli and Sontag^44^. To illustrate the key features of this map, we consider an example of a simple mutual activation feedback loop:

4 $$\frac{{dx}}{{dt}} = H\left( y \right) - x,\frac{{dy}}{{dt}} = H\left( x \right) - y,$$

where *H* is a Hill function (in fact, for our purposes, *H* can be any strictly monotonic function). Here, either variable is a size-one FVS, but we will consider *x* as the input/output variable:

5 $$\frac{{dx_{out}}}{{dt}} = H\left( y \right) - x_{out},\frac{{dy}}{{dt}} = H\left( {x_{in}} \right) - y,\frac{{dx_{in}}}{{dt}} = 0,$$

The characteristic feedback function $\kappa \left( x \right)$ is given by $\mathop {{\lim }}\limits_{t \to \infty } x_{out}$ for $x_{in}\left( 0 \right) = x$. Explicitly, $\kappa \left( x \right) = H\left( {H\left( x \right)} \right)$. The zeros of $\kappa \left( x \right) - x$ yield steady state values of *x* in the original equation. The slope of $\kappa \left( x \right) - x$ at these zeros describes the stability of the corresponding steady state; if the slope is negative, then the state is stable. The well-definition of *κ* in this example and in general relies on the fact that *x* forms an FVS. The stability properties rely on the sign-consistency of the underlying network.

This characteristic map method has been applied in the analysis of several biological systems, including the Lac Operon^47^, the Circadian Oscillator^48,49^, the MAPK Cascade Feedback^43^, and a classical model of testosterone concentration in the blood^50^; see Sontag^45^ for an in-depth discussion of these applications. Crucially, the feedback characteristic map can be experimentally measured more easily than individual interaction parameters^43^.

Of course, many biomolecular networks are not sign-consistent. It is usually a simple matter, however, to find subnetworks that are sign-consistent. As we have previously shown in^38,39^, it is possible to use these sign-consistent subnetworks to place bounds on the coordinate values of system trajectories that begin within specified subsets of the state space. These bounds can be used to identify trap sets in the dynamics, called stable motifs, that are robust to the effects of overriding unconstrained variables. This method has been successfully applied to several biological networks, including a T cell signaling network model, a *Drosophila* segment polarity model, and a cell cycle restriction switch model to identify robustly self-sustaining subcircuits^38,39,51^.

To illustrate how stable motifs, MIOS, and FVS methods build off one another, we consider a simple example modified from ref. ^39^

6 $$\begin{array}{l}\dot x = 1 - H\left( y \right) - x,\\\dot y = H\left( z \right)\left( {1 - H\left( x \right)} \right) - y,\\\dot z = 1 - \left( {1 - {\it{\epsilon }}} \right)H\left( {u\left( t \right)} \right) - z,\\H\left( \cdot \right) = \frac{{\left[ \cdot \right]^2}}{{\left[ \cdot \right]^2 + 1/16}}.\end{array}$$

**Fig. 2:**
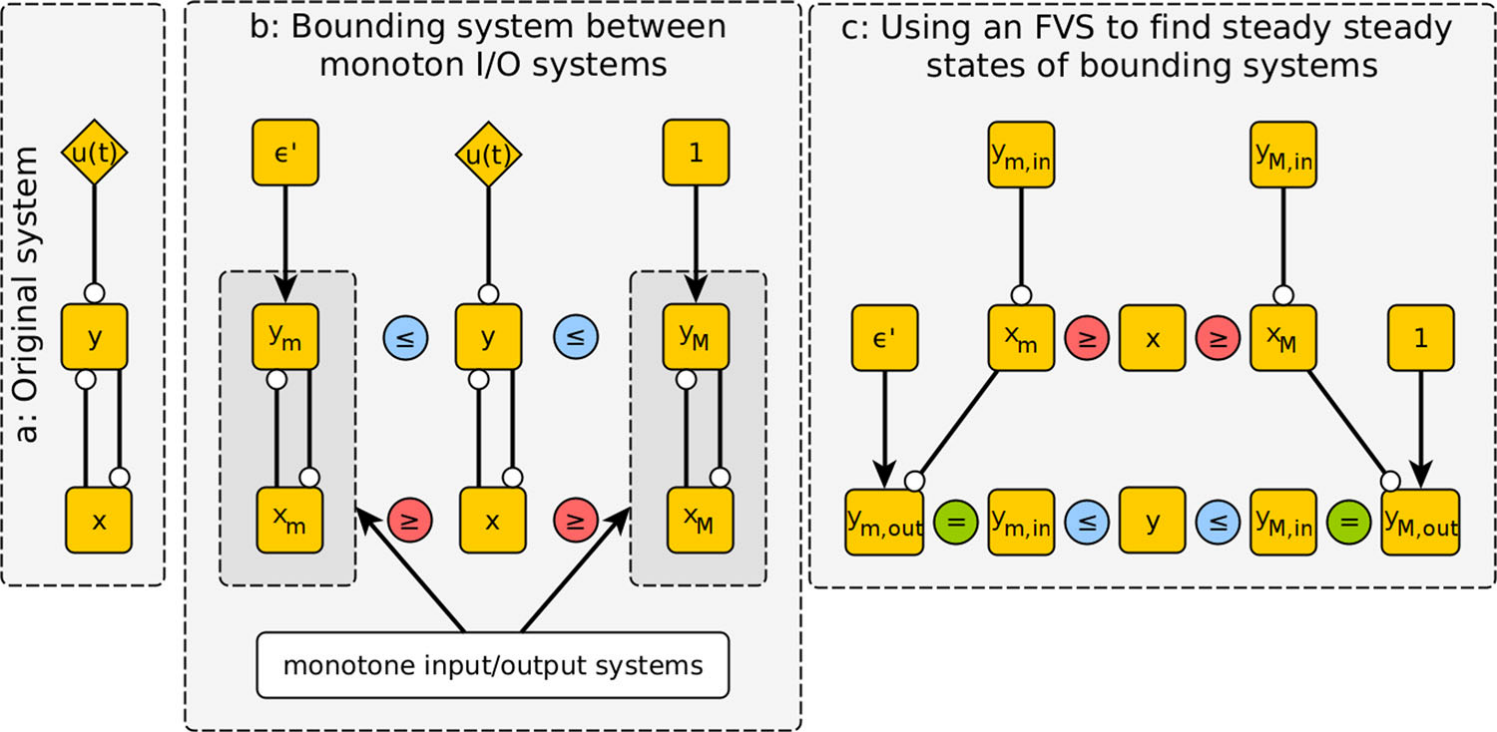
Schematic of stable motif procedure applied to the system of Eq. 6. The network structure of the system is depicted in panel **a**. Circle-tipped arrows represent inhibition, while wedge-tipped arrows represent activation. In panel **b**, the bounding systems in Eqs. 8 and 9 are depicted alongside the original system. Note that these bounding systems are monotone input/output systems. Panel **c** illustrates the characteristic feedback method of identifying steady states in monotone input/output systems, wherein the effect of overriding a feedback vertex set (FVS), in this case, the set containing $y_m$ or $y_M$, is considered, as described in the example of Eq. 4.

**Fig. 3:**
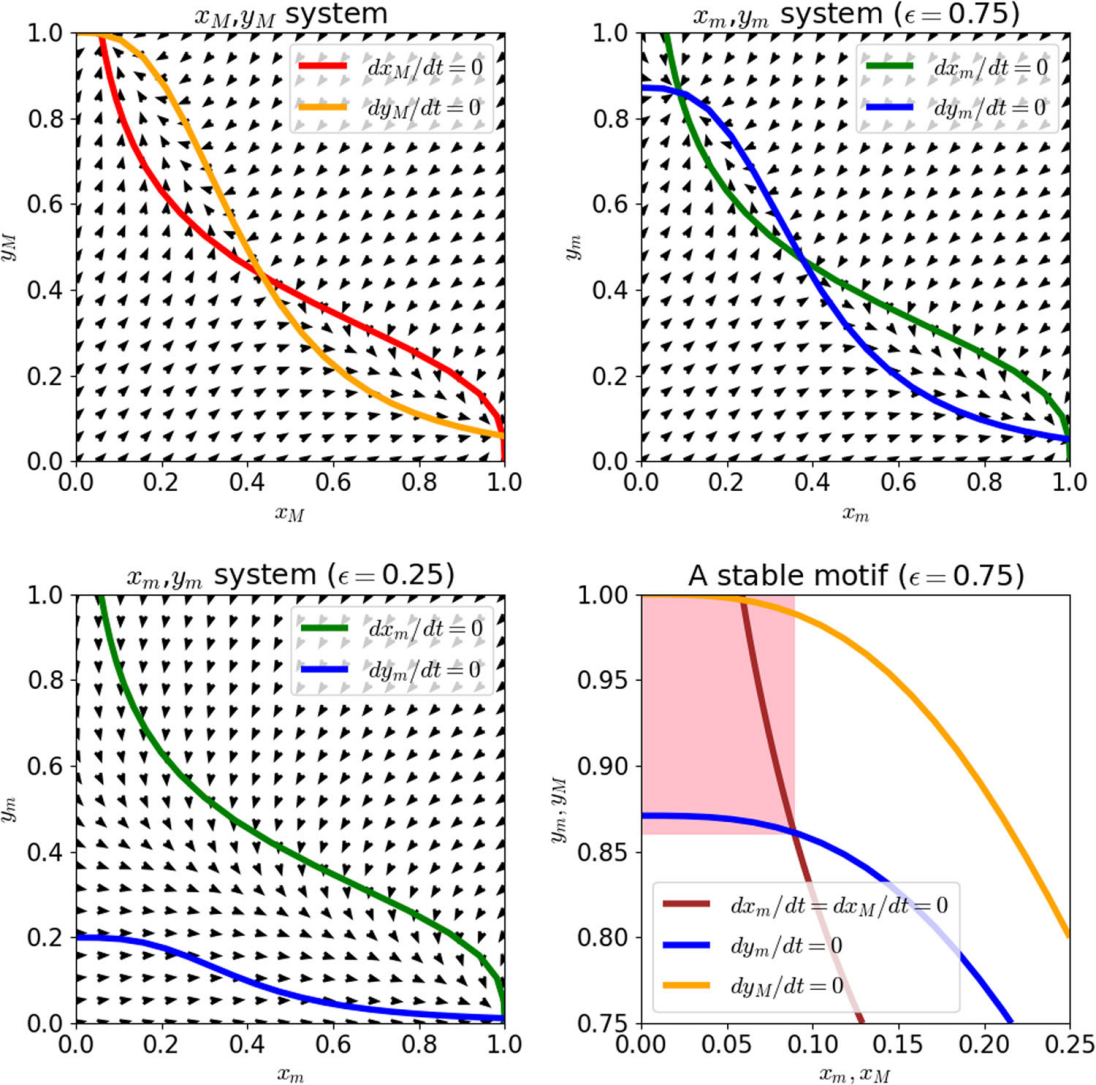
Phase portraits and nullclines of the bounding systems of Eqs. 8 and 9. The phase portrait of the bounding $x_M,y_M$ system is depicted in the top left. The trajectories of the *x,y* system are such that $x\left( t \right) \ge x_M\left( t \right)$ and $y\left( t \right) \le y_M\left( t \right)$. The phase portrait of the $x_m,y_m$ system is depicted in the top right and bottom left corners for two values of the parameter *ϵ*. For ${\it{\epsilon }} = 0.75$ (weak control strength), the system exhibits a steady state with small *x* and large *y*. Because $x_m\left( t \right) \ge x\left( t \right) \ge x_M\left( t \right)$ and $y_m\left( t \right) \le y\left( t \right) \le y_M\left( t \right)$ both hold, this steady state implies the existence of a subset of the state space that cannot be escaped by varying $u\left( t \right)$ in Eq. 6; this region is highlighted in red in the bottom right figure. Note that in the strong control case (${\it{\epsilon }} = 0.25$), there is no such steady state in the bounding system, and thus the control is able to drive the system from a low-*x*, high-*y* state to a high-*x*, low-*y* state.

Here, the mutual inhibition feedback loop between *x* and *y* is modulated by *z*, which is inhibited by y and controlled externally by *u* with strength $\left( {1 - {\it{\epsilon }}} \right) \in \left( {0,1} \right)$. In this example, we consider the behavior of the system in response to *u*. First, we note that *u* only affects *z* directly, and *z* depends only on *u*, so it is equivalent to consider the response of *x* and *y* in response to varying *z* between *ϵ* and *1*. Therefore, we set $z = 1 - \left( {1 - {\it{\epsilon }}} \right)H\left( {u\left( t \right)} \right)$ and consider

7 $$\dot y = H\left( {1 - \left( {1 - {\it{\epsilon }}} \right)H\left( {u\left( t \right)} \right)} \right)\left( {1 - H\left( x \right)} \right) - y$$

immediately, neglecting time delays that do not affect the ultimate behavior of *x* and *y*. The FVS formalism tells us that controlling *y* completely is enough to fully determine the behavior of *x*. It does not, however, tell us whether the particular control implementation here is sufficiently complete. The stable motifs method identifies that *x* and *y* form a monotone feedback loop subsystem for any fixed value of *z*. Because *z* is between ϵ and 1, this implies $H\left( {\it{\epsilon }} \right)\left( {1 - H\left( x \right)} \right) - y \,<\, \dot y \,<\, \left( {1 - H\left( x \right)} \right) - y$. Because the *x, y* subsystem is monotonic, $x_M\left( t \right) \le x\left( t \right) \le x_m\left( t \right)$ and $y_m\left( t \right) \le y\left( t \right) \le y_M\left( t \right)$ hold, where

8 $$\dot x_m = 1 - H\left( {y_m} \right) - x_m,\qquad\dot y_m = H\left( {\it{\epsilon }} \right)\left( {1 - H\left( {x_m} \right)} \right) - y_m,$$

9 $$\dot x_M = 1 - H\left( {y_M} \right) - x_M,\qquad\dot y_M = \left( {1 - H\left( {x_M} \right)} \right) - y_M,$$

for any initial conditions and any control input $u\left( t \right)$, provided that the inequalities $x_M\left( {t_0} \right) \le x\left( {t_0} \right) \le x_m\left( {t_0} \right)$ and $y_m\left( {t_0} \right) \le y\left( {t_0} \right) \le y_M\left( {t_0} \right)$ hold^39^, as can be proved by building on results presented by Angeli and Sontag^41–43^. The implication is that any steady states $\bar x_m,\bar y_m$ and $\bar x_M,\bar y_M$ of the $x_m,y_m$ and $y_M,y_M$ systems, respectively, describe forward time-invariant trap sets in the dynamics that are independent of the control input. This procedure is illustrated in Fig. 2. For example, with a control strength of 1⁄4 (${\it{\epsilon }} = 0.75$), it is straightforward to show (see Supplementary code) that the $x_m,y_m$ system has a steady state with $\bar x_m$ between 0.089 and 0.090 and$,\bar y_m$ between 0.861 and 0.862. This means that trajectories whose initial conditions satisfy $x\left( {t_0} \right) \le 0.090$ and $y\left( {t_0} \right) \ge 0.862$ cannot be controlled to a state in which $x\left( {t_f} \right) \ge 0.089$ or $y\left( {t_f} \right) \le 0.861$; this trap space corresponds to the robust feedback loop, or stable motif, between *x* and *y*, and is highlighted in Fig. 3. The existence of such a trap space demonstrates that the switch remains robustly bistable when subjected to such a weak control. This is not the case in the high-strength limit ${\it{\epsilon }} \to 0$, because large $u\left( t \right)$ causes $y\left( t \right)$ to exponentially approach zero. We can see from Fig. 3 that at ${\it{\epsilon }} = 0.25$, the stable motif has been destroyed, allowing for the control signal to steer the system to either of the two qualitative switch states.

In this example, we have calculated the relevant trap set explicitly and quantitatively, though this is not always necessary. In fact, the stable motif trap set approach is a generalization of methods devised for the study of Boolean networks^52–55^. We have shown in earlier work^51^ that this connection can be exploited to evaluate the sensitivity of control strategies to changes in parameter values. Additional connections between trap set methods and Boolean analysis have been further discussed elsewhere by us^38,39^ and by Schwieger et al. ^56^.

This example illustrates that limited control over a feedback vertex set (in this case, *y*) is often not sufficient to guarantee convergence to a desired attractor. Here, *y* is controlled via an external signal, $u\left( t \right)$, which is mediated by a variable *z*. Whether this mediator variable is viewed as a dynamical variable or an algebraic quantity, the fact remains that the signal is not sufficient to directly force *y* to take the values attained in the system’s attractors. In some cases, full attractor control is nonetheless possible, as we have seen when *ϵ* is low. In other cases, as illustrated for ${\it{\epsilon }} = 0.75$, the limited control over the FVS is insufficient to drive the system out of certain control-robust trap sets.

## Discussion

Complex networks are a common tool in the modeling repertoire of biologists, and describe the intricate webs of interactions that underlie important processes of biological and medical interest. ODEs are frequently employed to understand the dynamics these networks describe. It is of key interest to understand how to drive these dynamical systems to or away from target configurations. This task has applications in the identification of drug targets, oncogenes, and signaling pathways, and helps provide insights into the functional role of biomolecular subcircuits. Unfortunately, this task is complicated by the high dimension in which these dynamical systems typically reside, as well as by poor constraints on model parameters. It is also important when seeking to apply control insights in practice to consider that interventions suggested by classical control theory often assume that the system under analysis can be engineered to a greater degree than may be practical in biomolecular systems. Indeed, experimental probes and medical interventions (e.g., drugs) in biomolecular networks often take the form of an “all or nothing” effect on an individual mRNA or protein. Constructing intricate time-varying controls has seen some success in carefully constructed experiments, but this level of control is often impractical.

These challenges make desirable those methods that emphasize nonperturbative control and identification of network structure (for which data is more readily available than for rate parameters). When compared to a state-space point of view, a network-centric point of view scales very well with the dimension of a system. In this review, we have highlighted several methods that exploit the role network structure plays in constraining the dynamics of a system. These methods help to illuminate those properties of a dynamical biological system that are not sensitive to the particular choice of functional form or parameter values. They exploit the robustness of the conclusions one can draw from the network topology alone to make mathematical conclusions that are more readily translated into practice. They demonstrate a key mathematical insight that has been uncovered through the study of biological systems: the network of interactions between entities in a complex system is a fundamental determinant of possible emergent behaviors. It is our hope that continued exploration of the connections between network structure and dynamics will bring ever deeper understanding of how complexity arises from the simple rules that govern biomolecular interactions at the cellular level.

## Supplementary information

Supplementary Code

Supplementary Notebook

Supplementary Notebook (PDF)

## Data Availability

All data used in this review are included in this manuscript or are available as supplementary material.

## Code availability

All software created for this review are available as supplementary material.
