# Supplementary material for: Leveraging network structure in nonlinear control
Source: NPJ Syst Biol Appl. 2022 Oct 1;8:36. doi: 10.1038/s41540-022-00249-2 (PMC9526710; doi:10.1038/s41540-022-00249-2)
Supplement: Supplementary file 3 — Supplementary Notebook (PDF) [file 41540_2022_249_MOESM3_ESM.pdf]

Calculations for the example of equations 2 and 3

```
from scipy.optimize import fsolve
import autograd.numpy as np
import autograd as ag
```

Define the equations; EQ corresponds to equation 2, and eq corresponds to equation 3.

```
n = 4
kn = 1/8**n

def H(x):
    xn = x**n
    return xn/(xn+kn)

def EQ(P):
    X,Y,Z=P
    dX = H(Y/3 + Z/3) - X
    dY = 3*H(X)*H(Z-X) + H(Y/3 + Z/3) - Y
    dZ = 2*H(Y/3 + Z/3) - Z
    return np.array([dX,dY,dZ])

def eq(p):
    x,y,z=p
    dx = H(y) - x
    dy = H(x)*H(z) + H(y) - y
    dz = H(y) - z
    return np.array([dx,dy,dz])
```

Calculate the steady states of equation 2, and verify that two are stable and one is unstable by linearlization

```
Xf,Yf,Zf=fsolve(EQ,(1,4,2))
JAC=ag.jacobian(EQ)(np.array([Xf,Yf,Zf]))
print("point",(Xf,Yf,Zf))
print("error",EQ((Xf,Yf,Zf)))
print("eigenvalues",np.linalg.eig(JAC)[0])

Xf,Yf,Zf=fsolve(EQ,(0,0,0))
JAC=ag.jacobian(EQ)(np.array([Xf,Yf,Zf]))
print("\npoint",(Xf,Yf,Zf))
print("error",EQ((Xf,Yf,Zf)))
print("eigenvalues",np.linalg.eig(JAC)[0])
```

```
Xf,Yf,Zf=fsolve(EQ,(0.06,0.07,0.12))
JAC=ag.jacobian(EQ)(np.array([Xf,Yf,Zf]))
print("\npoint",(Xf,Yf,Zf))
print("error",EQ((Xf,Yf,Zf)))
print("eigenvalues",np.linalg.eig(JAC)[0])
```

```
point (0.999984726071823, 3.998520329155487, 1.999969452143646)
error [-1.11022302e-16  0.00000000e+00 -2.22044605e-16]
eigenvalues [-1.         -1.00022941 -0.99974003]

point (0.0, 0.0, 0.0)
error [0. 0. 0.]
eigenvalues [-1. -1. -1.]

point (0.060275496139729566, 0.06816831264137221, 0.12055099227945913)
error [-9.89000548e-14 -2.28736474e-12 -1.97800110e-13]
eigenvalues [-1.         -1.30538439  2.90707384]
```

Repeat the procedure for equation 3

```
xf,yf,zf=fsolve(eq,(1,2,1))
jac=ag.jacobian(eq)(np.array([xf,yf,zf]))
print("point",(xf,yf,zf))
print("error",eq((xf,yf,zf)))
print("eigenvalues",np.linalg.eig(jac)[0])

xf,yf,zf=fsolve(eq,(0,0,0))
jac=ag.jacobian(eq)(np.array([xf,yf,zf]))
print("\npoint",(xf,yf,zf))
print("error",eq((xf,yf,zf)))
print("eigenvalues",np.linalg.eig(jac)[0])

xf,yf,zf=fsolve(eq,(0.06,0.06,0.06))
jac=ag.jacobian(eq)(np.array([xf,yf,zf]))
print("\npoint",(xf,yf,zf))
print("error",eq((xf,yf,zf)))
print("eigenvalues",np.linalg.eig(jac)[0])
```

```
point (0.9999847260718229, 1.9994965937663778, 0.9999847260718229)
error [ 0.00000000e+00 -2.22044605e-16  0.00000000e+00]
eigenvalues [-0.99974003 -1.         -1.00022941]

point (0.0, 0.0, 0.0)
error [0. 0. 0.]
eigenvalues [-1. -1. -1.]

point (0.06027549613980553, 0.06290643497414032, 0.06027549613980553)
```

```
error [1.73355080e-12 5.75331449e-13 1.73355080e-12]
eigenvalues [ 2.90707384 -1.         -1.30538439]
```

Define modified versions equation 2 where each of the three genes have been knocked out (i.e., the corresponding variable is forced to be zero)

```
def EQ_Z(P):
    X=P[0]
    Y=P[1]
    dX,dY,dZ=EQ((X,Y,0))
    return np.array([dX,dY])

def EQ_Y(P):
    X=P[0]
    Z=P[1]
    dX,dY,dZ=EQ((X,0,Z))
    return np.array([dX,dZ])

def EQ_X(P):
    Y=P[0]
    Z=P[1]
    dX,dY,dZ=EQ((0,Y,Z))
    return np.array([dY,dZ])
```

Verify that none of these knockouts is alone sufficient to drive the system away from the "high activity" stable fixed point, i.e., the 2-dimensional knockout systems each retain a "high activity" stable fixed point

```
Xf,Yf=fsolve(EQ_Z,(1,4))
JAC=ag.jacobian(EQ_Z)(np.array([Xf,Yf]))
print("point",(Xf,Yf))
print("error",EQ_Z((Xf,Yf)))
print("eigenvalues",np.linalg.eig(JAC)[0])

Xf,Zf=fsolve(EQ_Y,(1,2))
JAC=ag.jacobian(EQ_Y)(np.array([Xf,Zf]))
print("\npoint",(Xf,Zf))
print("error",EQ_Y((Xf,Zf)))
print("eigenvalues",np.linalg.eig(JAC)[0])

Yf,Zf=fsolve(EQ_X,(4,2))
JAC=ag.jacobian(EQ_X)(np.array([Yf,Zf]))
print("\npoint",(Yf,Zf))
print("error",EQ_X((Yf,Zf)))
```

```
print("eigenvalues",np.linalg.eig(JAC)[0])
```

```
point (0.9999226391254272, 3.998457878600556)
error [0. 0.]
eigenvalues [-1.00063567 -0.99928694]

point (0.9987594267453773, 1.9975188534907546)
error [-1.11022302e-16 -2.22044605e-16]
eigenvalues [-1.         -0.99503771]

point (0.9997556803424631, 1.9995113606849262)
error [0. 0.]
eigenvalues [-1.         -0.99902272]
```

Construct the version of equation 3 in which y has been knocked out

```
def eq_y(p):
    x=p[0]
    z=p[1]
    dx,dy,dz=eq((x,0,z))
    return np.array([dx,dz])
```

Verify the prediction of the FVS method; i.e., that knocking out variable y in equation 3 is alone sufficient to drive the system to the "low activity state" from the "high activity" state

```
xf,zf=fsolve(eq_y,(1,1))
jac=ag.jacobian(eq_y)(np.array([xf,zf]))
print("\npoint",(xf,zf))
print("error",eq_y((xf,zf)))
print("eigenvalues",np.linalg.eig(jac)[0])
```

```
point (0.0, 0.0)
error [0. 0.]
eigenvalues [-1. -1.]
```
